# Tetrodotoxin-Resistant Sodium Channels Contribute to Directional Responses in Starburst Amacrine Cells

**DOI:** 10.1371/journal.pone.0012447

**Published:** 2010-08-27

**Authors:** Nicholas W. Oesch, W. Rowland Taylor

**Affiliations:** 1 Neuroscience Graduate Program, Oregon Health and Sciences University, Portland, Oregon, United States of America; 2 Casey Eye Institute, Oregon Health and Sciences University, Portland, Oregon, United States of America; Lund University, Sweden

## Abstract

The biophysical mechanisms that give rise to direction selectivity in the retina remain uncertain. Current evidence suggests that the directional signal first arises within the dendrites of starburst amacrine cells (SBACs). Two models have been proposed to explain this phenomenon, one based on mutual inhibitory interactions between SBACs, and the other positing an intrinsic dendritic mechanism requiring a voltage-gradient depolarizing towards the dendritic tips. We tested these models by recording current and voltage responses to visual stimuli in SBACs. In agreement with previous work, we found that the excitatory currents in the SBACs were directional, and remained directional when GABA receptors were blocked. Contrary to the mutual-inhibitory model, stimuli that produce strong directional signals in ganglion cells failed to reveal a significant inhibitory input to SBACs. Suppression of the tonic excitatory conductance, proposed to generate the dendritic voltage-gradient required for the dendrite autonomous model, failed to eliminate the directional signal in SBACs. However, selective block of tetrodotoxin-resistant sodium channels did reduce the strength of the directional excitatory signal in the SBACs. These results indicate that current models of direction-selectivity in the SBACs are inadequate, and suggest that voltage-gated excitatory channels, specifically tetrodotoxin-resistant sodium channels, are important elements in directional signaling. This is the first physiological evidence that tetrodotoxin-resistant sodium channels play a role in retinal information processing.

## Introduction

Direction selective ganglion cells (DSGCs) in the retina require GABAergic inhibition, mediated by GABA_A_ receptors, for the discrimination of motion direction [1], [2], [3], [4]. The GABAergic input to DSGCs is two to ten times larger for null direction motion than for preferred direction motion [5], [6], [7], [8], suggesting that directional inhibition is a key mechanism generating directional responses. The most likely source for this directional inhibition is the SBAC, a radially symmetrical, inhibitory interneuron that is both GABAergic and cholinergic [7], [9], [10]. It has been suggested that the SBAC dendrites are the first point where directional signals in the retina arise.

In support of this notion, SBAC dendritic varicosities display large calcium transients during centrifugal stimulation, when the stimulus moves from the cell soma towards the tips of the dendrites, whereas calcium transients are small or absent during centripetal stimulation, when the stimulus moves towards the soma [11], [12], [13]. Similarly, voltage responses measured at the soma are larger for centrifugal motion than for centripetal motion and these differences in the voltage responses presumably drive the differences in calcium signals.

Recently, two mechanisms have been proposed to explain how the directional asymmetries in the voltage responses and calcium transients of SBACs arise. Lee & Zhou [12] demonstrated that neighboring SBACs make reciprocal GABAergic contacts and suggested that an anatomical asymmetry in these contacts generates inhibition in the SBAC that is larger for centripetal than centrifugal motion. They found that inhibition suppressed the voltage and calcium signals during centripetal motion originating outside the extent of the dendritic arbor. In contrast, Hausselt *et al.* [13] demonstrated that directional voltage signals in the SBAC were generated when the stimulus was restricted to the dendritic arbor of a single SBAC, arguing that the involvement of neighboring SBACs was not necessary. Furthermore, they found that GABA_A_ receptor antagonists had no effect on the directional calcium or voltage signals they recorded [13] and they proposed that directional signals were due to sequential activation of excitatory inputs to SBACs superimposed on an asymmetrical voltage gradient in the dendrites generated by tonic AMPA receptor mediated synaptic current acting in concert with voltage-gated calcium channels.

These two proposed mechanisms are quite different, but not mutually exclusive. Thus, while it is generally agreed that the origin of direction selective signals lies within the SBAC, the precise biophysical mechanisms giving rise to direction selectivity remain elusive. In this report, we examined the directional synaptic responses in the SBACs and tested key components of the two proposed models and found that neither model is adequate to explain the existence of directional signals in the SBAC.

## Methods

All animal procedures were conducted in accordance with NIH guidelines and protocol number 0735 approved by the Institutional Animal Care and Use Committee at OHSU.

### Tissue Preparation and Maintenance

Dark-adapted, out-bread pigmented rabbits were surgically anesthetized using intra-muscular 60 mg/kg Ketamine and 10 mg/kg xylazine and i.v. sodium pentobarbital (40 mg/kg) and the right eye removed under dim-red illumination (>620 nm). Following enucleation, animals were euthanized by anesthetic overdose of i.v. sodium pentobarbital, followed by i.v. injection of 10 ml of 3M KCl. All subsequent manipulations were performed under infrared illumination (>900 nm). The anterior portion of the eye was removed, the eyecup transected just above the visual streak, and the dorsal piece discarded. The retina was dissected from the sclera, and a 5×5 mm section of central retina was adhered, photoreceptor-side down, to a glass cover-slip coated with poly-L-lysine (Sigma) or Cell-Tak (Becton Dickinson GmbH, Germany). The preparation was placed in a recording chamber (∼0.5 ml volume) and continually perfused (∼4 ml/min) with oxygenated bicarbonate-buffered Ames medium [14], pH 7.4 maintained at 34–36°C. The major electrolytes in Ames medium are (mM): 120 NaCl, 23 NaHCO_3_, 3.1 KCl, 1.15 CaCl_2_ and 1.24 MgCl_2_. Pharmacological agents were dissolved in oxygenated Ames medium and perfused through the recording chamber identically to the control Ames solution.

### Electrophysiology and Light Stimulation

Patch electrodes were pulled from borosilicate glass to have a final resistance of 4–8 MΩ. For extracellular recording, the electrodes were filled with Ames medium. For voltage-clamp recording, the electrodes were filled with the following electrolytes: 110 mM Cs-methylsulfonate, 10 mM NaCl, 5 mM Na-HEPES, 1 mM EGTA, 1 mM Na-ATP, 0.1 mM Na-GTP. For current clamp recordings K-methylsulfonate was substituted for Cs- methylsulfonate. Retinal neurons were visualized using differential infrared differential interference contrast microscopy or Dodt optics [15]. ON starburst amacrine cells (SBACs) were targeted based on their location in the ganglion cell layer and their comparatively small round somas. Prior to patching the SBAC we made a small hole in the inner limiting membrane near the cell soma, through which the patch electrode was applied to the cell. The SBACs identity was confirmed by its physiological response characteristics, which included a high variance noise consisting of large fast inward currents, a biphasic light response with a fast transient component, a sustained component, and a characteristic decrease in noise at light termination. For ganglion cells we targeted cells with a medium soma diameter and a crescent-shaped nucleus [16]. The extracellular electrode was applied to the soma under visual control through a small hole in the overlying inner limiting membrane, and a loose patch recording was formed. After establishing that the ganglion cell was a DSGC and determining its preferred direction (see below), the extracellular recording electrode was removed and an intracellular patch-electrode applied to the same cell. During whole cell recordings current signals were filtered at 4 kHz through the 4-pole Bessel filter of the EPC10 dual amplifier (HEKA Elektronics, Canada), and digitized at 10–50 kHz.

Light stimuli, generated on a mini-CRT computer monitor (MicroBrightField, Inc, refresh rate, 60 Hz), were focused onto the photoreceptor outer segments through a 20× (NA 0.95) Olympus water-immersion objective. The percent stimulus contrast was defined as C = 100×(L−L*_mean_*)/L*_mean_*, where L is the stimulus intensity and *L_mean_* is the background intensity. For SBAC stimulation the standard moving stimulus consisted of two 150×900 µm bars moving along their long axis in opposite directions at 900 µm/s, with each bar stimulating an opposing half of the SBAC dendritic field. The bars enter and exit the stimulus field from behind a mask with a rectangular aperture 150 µm wide and 680 µm long with the long axis oriented parallel to the direction of motion. The short edge of the aperture was offset 20 µm from the centre of the soma, and therefore the opposite edge was 680+20 µm distant from the soma. Thus, for centrifugal motion the edges of the rectangle first appear from behind the mask 20 µm from the center of the soma, and disappeared 700 µm from the soma. Because the bar is 900 µm on its long axis and moves at 900 µm/s the leading and trailing edges were separated in time by 1 s. For centripetal motion the stimulus direction was simply reversed. The contrast of the bar was set to 100%. For DSGCs the standard moving stimulus comprised a light or dark bar, moving along its long axis at 800–1200 µm/s on the retina, and traversed the entire width of the stimulation field. The bar's width was 250 µm, and its length was set to achieve a 1–2 second separation of the leading and trailing edge responses. All light stimuli were centered with respect to the tip of the recording electrode, and thus also with the soma of the target cell. The stimulus area was limited by the size of the CRT monitor, and covered a square region 0.7 mm on a side. Since the dendritic arbors of SBAC and DSGCs range from 200 µm to 400 µm for SBACs [17], [18] 300 µm to 400 µm for DSGCs [16], they were fully contained within the stimulus area. Data acquisition was triggered at a fixed time relative to the light stimulus and therefore it was straight-forward to calculate the position of the stimulus at any time during the response.

### Analysis

Analysis was performed using custom procedures in IgorPro (Wavemetrics, USA). For SBACs, the magnitude of the directional asymmetry was calculated using an asymmetry index (AI), which ranged from 0 to 1, with values closer to one indicating a stronger asymmetry. For voltage and current measurements we defined AI = (centrifugal−centripetal)/( centrifugal+centripetal), so that a positive index indicates that centrifugal motion is preferred. For the rise time, the AI was defined as AI = (centripetal−centrifugal)/(centrifugal+centripetal), so that a positive index indicates that centrifugal motion is faster. For DSGCs, the preferred direction of the cells and the strength of the directional tuning were calculated from responses to stimuli in each of 12 stimulus directions evenly spanning 360 degrees. Responses were represented as vectors with the angle representing the direction of stimulus motion, and length equal to the number of action potentials or the peak amplitude of PSPs. The preferred direction was obtained from the angle of the resultant vector, which was the vector sum of all 12 responses. The directional tuning index (DSI) was calculated as the normalized length of the resultant vector. DSI can range from 0, when the magnitude of the response is the same in all stimulus directions, to 1, for perfect tuning when a response is produced only for a single stimulus direction [6]. The directional tuning data is well described by a von Mises distribution, which is the circular analogue to the Gaussian distribution. The response *R*, as a function of stimulus direction is given as, *R = R_max_ e^(κ cos((x−μ)π/180))^/e^κ^*, where *R_max_* is the maximum response, *μ* becomes the preferred direction in degrees, and κ is the concentration parameter, which accounts for the width of the directional tuning.

Conductance was calculated using methods previously described [6], [8]. Briefly, light evoked synaptic currents were assumed to arise from a sum of linear excitatory and inhibitory synaptic inputs. Excitation is mediated by non-selective cation channels with reversal potential, *V_e_* = 0 mV, while inhibition is mediated by chloride channels with reversal potential, *V_i_* = −69 mV. Reversal potentials were calculated from our internal and external recording solutions. The synaptic currents obey Ohm's law so that *I_e_ = g_e_(t)(V−V_e_)*, and *I_i_ = g_i_(t)(V−V_i_)*, where the inhibitory, *g_i_(t)* and excitatory, *g_e_(t)* conductances are functions of time. The total light evoked synaptic current is, *I_T_ = g_T_(t)(V−V_t_(t))*, where *g_T_ = g_e_+g_i_*. The observed synaptic reversal potential *V_r_(t)* is a weighted sum of *V_e_* and *V_i_* such that, *V_r_(t) = (g_e_(t )/g_T_(t))V_e_+(g_i_(t)/g_T_(t))V_i_*. The excitatory and inhibitory synaptic conductances can be calculated from *g_T_(t)* and *V_r_(t)* according to the equations: *g_e_(t) = g_T_(t)(V_r_(t)−V_i_)/(V_e_−V_i_)* and *g_i_(t) = g_T_(t)(V_r_(t)−V_e_)/(V_i_−V_e_)*. To collect the data necessary to calculate the synaptic conductance we presented centrifugal or centripetal (or preferred or null) stimuli while holding the cell at command voltages ranging between −100 mV and 0 mV by increments of 20 mV. We then calculated IV relations for the net light-evoked currents every 10 ms. Each IV was fit with a line between −100 mV and −40 mV where the IV relation was most linear. The slope *g_T_* and intercept *V_r_* were determined from the fit, thus producing a discrete measurement for *g_T_* and *V_r_* at every point.

To examine voltage-activated currents in the SBACs we applied a family of command voltages between −100 mV and 0 mV by increments of 20 mV. To isolate the voltage-activated component of the membrane current, we subtracted the linear leak current measured between −100 mV and −40 mV current from the IV measured over the full voltage range.

Unless otherwise noted, the mean ± standard deviation is quoted throughout the paper. Significance values for comparing pharmacological manipulations to control were generated using the Students paired t-test, significance was considered to be p<0.05.

## Results

**Figure 1:**
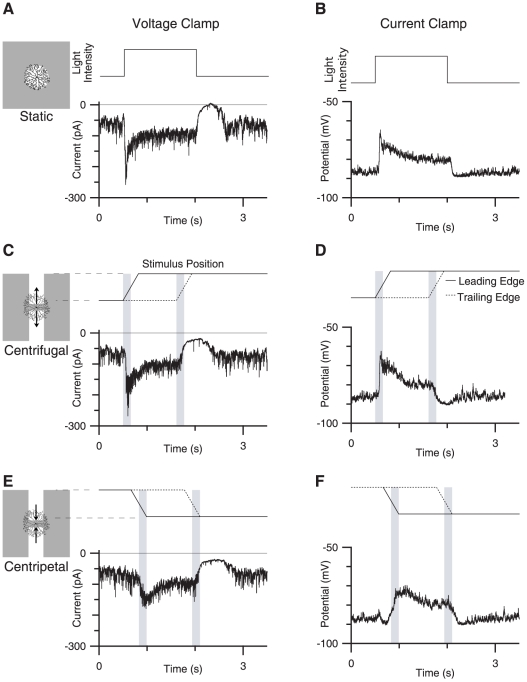
Voltage and current responses to static and moving stimuli. A, Currents recorded in response to a 200 µm spot of light centered over the receptive field against a gray background, contrast 100%. B, Voltage response to an identical stimulus. C and E show currents in response to centrifugal and centripetal moving stimuli, respectively (see Methods). The solid and dotted lines above the trace indicates the position along the long axis of motion of the leading and trailing edge of the stimuli on the y-axis plotted against time. (see Methods). The shaded area over the traces represents the time when the leading and trailing edges of the stimuli are estimated to be moving over the cells dendritic arbor. D and F, show voltage responses to the same stimuli shown in C and E. The inset to the left is a schematic representation of a SBAC and the stimulus field showing the scale of the stimuli relative to a representative SBAC, the stimulus field denoted by the shaded square is 700 µm on a side.

To examine the synaptic processes that mediate directional signaling in the starburst amacrine cell (SBAC), we made whole-cell voltage-clamp and current-clamp recordings from ON SBACs in isolated whole-mount rabbit retina, and stimulated the retinal circuitry with patterns of light projected onto the retinal photoreceptors (see methods). In response to a 200 µm diameter light-flash centered on the cell soma, the SBAC produced a peak response of 117±7 pA with a 10% to 90% activation time of 30±10 ms (Figure 1A). In accordance with previous work, the peak response was transient and decayed to a sustained inward current for the duration of the light stimulus (1.5 s) [12], [17], [18]. At the termination of the light flash there was a marked suppression of a tonic inward current, and a concomitant decrease in the membrane current variance. Current clamp recordings closely mirror the current response, suggesting that the observed excitatory currents predominantly drive the voltage response (Figure 1B).

### SBACs respond differentially to centrifugal and centripetal motion

To study the asymmetric response between centrifugal and centripetal motion, we stimulated the SBAC by drifting two opposing rectangles away from (centrifugal stimulation), or towards (centripetal stimulation) the soma (Figure 1C & E). We chose this stimulation paradigm because such stimuli elicit robust directional responses from DSGCs [5], [6], [7], [19], [20].

The responses to moving stimuli were qualitatively similar to the flash responses shown in Fig 1A; there is a transient inward current as the stimulus enters the receptive field, a sustained inward current while the light intensity remains elevated, and an outward current with a concomitant reduction in current noise levels as the trailing edge of the stimulus exits the receptive field. The most prominent asymmetry between centrifugal and centripetal stimulation was found in the initial transient response, which was smaller for centripetal stimulation. For centrifugal motion the peak amplitude of the response was −103±30 pA and the 10–90% rise-time was 38±7 ms (n = 30). For the corresponding centripetal motion the peak amplitude was −66±22 pA and the 10–90% rise time was 196±83 ms. Both peak amplitude and rise time were significantly different between centrifugal and centripetal motion (p<0.001, n = 30).

Thus, during centripetal motion the peak amplitude was reduced by 36% relative to centrifugal motion, while the rise-time increased by 515%. These values correspond to asymmetry indices (AIs, see Methods) of 0.22±0.07 and 0.67±0.14 for amplitude and rise time respectively. The directional asymmetry observed here, characterized by larger, faster responses for centrifugal motion, are qualitatively similar to previous observations [11], [12], [13]. Current-clamp recordings indicated that the directional asymmetry of the voltage response is also similar to the asymmetry observed for the currents (Figure 1, AI = 0.175±0.064 for amplitude and 0.634±0.059 for rise time, n = 4).

Calculation of the total charge transferred during the time while the leading edge of the stimulus traversed the cell's receptive field, indicates that the SBAC receives a greater amount of excitation during centrifugal stimulation relative to centripetal stimulation. The total charge was 24.0±8.7 pC for centrifugal motion versus 12.5±4.5 pC for centripetal motion (p<0.001, n = 30). This difference corresponded to an AI for the current-integral of 0.331±0.137. Thus, both the waveform of the currents and the total amount of charge transfer is directional.

### Excitatory conductance in the SBAC is directional, but inhibition is absent

**Figure 2:**
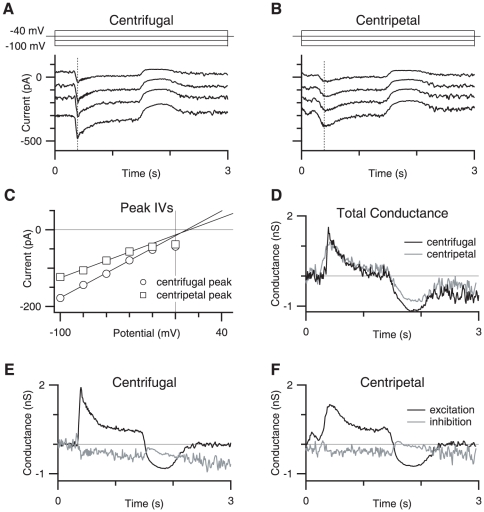
Conductance measurements of SBAC synaptic inputs. A,B, Currents recorded during centrifugal and centripetal stimulation, respectively during command potentials ranging from −100 mV to −40 mV (traces filtered, 25 Hz Gaussian). Schematic above shows command voltage. Vertical dotted lines indicate the time point at which sample IVs shown in C were measured. C shows plots of IV at the peak responses as indicated by the dotted line in figure A and B for centrifugal (circles symbols) and centripetal (square symbols) motion. The solid line indicates the linear fit between −100 mV and −40 mV. D, Total conductance measured every 10 ms for centrifugal and centripetal traces, in black and gray, respectively. E,F, Excitatory (black traces) and inhibitory conductances (gray traces) for centrifugal and centripetal motion, respectively.

Lee and Zhou, [12] demonstrated that GABAergic inhibition in response to stimulation outside the dendritic extent of the SBAC shaped the voltage response to centripetal stimulation. To examine the role of inhibition, we performed whole-cell voltage clamp recordings and measured the excitatory and inhibitory conductance during centrifugal and centripetal stimuli over a range of holding potentials (Figure 2 and see methods). Current-voltage relations, measured near the peak of the response, (dotted lines Figure 2A &B) were linear for both centrifugal and centripetal stimulation (Figure 2C, see [17]). Comparing the slopes of the I-V relations indicates that the peak light-evoked conductance is larger during centrifugal stimulation (Figure 2D). We calculated the excitatory and inhibitory components of the conductance based on a reversal potential of 0 mV for excitation and −69 mV for inhibition (Figure 2E & F, see Methods).

**Figure 3:**
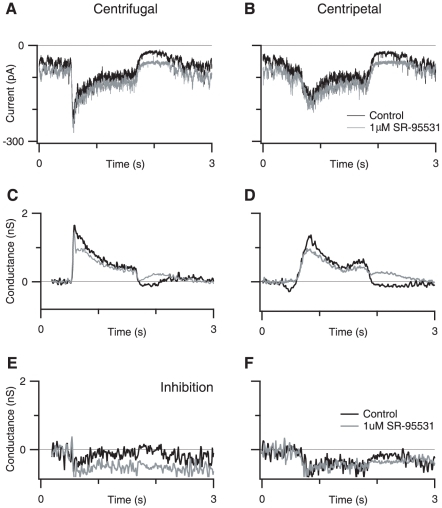
GABAergic inhibition does not contribute to responses to moving stimuli. A,B, Control currents (black traces) and currents recorded in the presence of 1 µM SR-95531 (gray traces), in response to centrifugal (A) and centripetal stimuli (B). C,D: excitatory conductance for centrifugal (C) and centripetal (D) stimulation in control (black traces) and SR-95531 (gray traces). E,F, Inhibitory conductance for centrifugal (E) and centripetal (F) stimulation in control (black traces) and SR-95531 (gray traces).

These measurements revealed that essentially all the current was due to the activation of excitatory synaptic inputs, suggesting that inhibitory synaptic input did not contribute significantly, if at all, to the responses. Due to the possibility that the SBAC is electrotonically large, we pharmacologically confirmed that inhibitory receptors do not contribute to differences between centrifugal and centripetal stimulation by applying the GABA_A_ receptor antagonist SR-95531 (1 µM) to block inhibitory synaptic input (Figure 3A,B). SR-95531 did not alter the amplitude, integral, or rise time of the current responses, or conductance measurements (Figure 3C,D). Nor was there a significant change in the directionality for any of these metrics (n = 5). Taken together, the conductance measurements (Figure 2) and the results from blocking GABAergic transmission (Figure 3), indicate that inhibition does not shape the directional signals recorded at the soma. Because inhibition does not appear to contribute to the SBAC responses we will continue to report measurements of directional differences taken from the measured inward currents, as this provides a more direct measurement of the cell response compared to the calculated conductance.

### NBQX blocks direction selectivity by blocking excitation in SBACs

Even in the absence of GABAergic input to the SBAC it is clear that centrifugal and centripetal responses remain directional (Figure 3C). Hausselt *et al.* [13] observed similar results, and proposed that a proximal to distal voltage gradient in the SBAC dendrite created an electrical asymmetry between centrifugal and centripetal stimulation, which acts in concert with nonlinear conductances to generate directional responses. They proposed that this voltage gradient was generated by a tonic excitatory input to the SBAC dendrites [17], [18]. Consistent with this theory, previous groups have reported that quinoxaline AMPA/kainate antagonists (CNQX, NBQX) block the tonic input to SBACs [13], [17], [18], and eliminate directional responses in the DSGC [21]. However, Cohen & Miller [21] proposed that the loss of directional selective DS responses in DSGCs in the presence of quinoxalines was due to suppression of the excitatory drive to starburst amacrine cells, with the result that both GABA and acetylcholine release from these cells was blocked. To determine the mechanism of NBQX block of direction selectivity we measured the effects of NBQX on synaptic conductances in SBACs.

**Figure 4:**
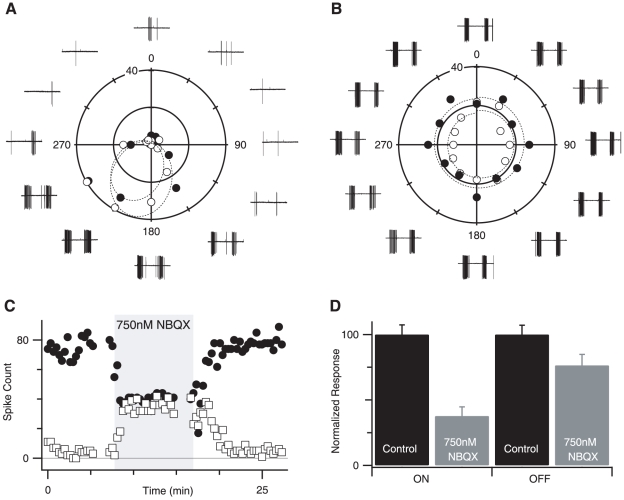
NBQX blocks direction selectivity in DSGCs. A, Extracellular spike responses to sweeping bar stimuli in each of 12 directions are shown adjacent to the stimulus angle. Each point on the polar plot shows the total number of spikes for the ON (open symbols) and OFF (closed symbols). The solid lines indicate the best-fit von Mises distribution for the ON and OFF responses. Asymmetry in polar plot indicates directional selectivity of the response. B, Same as A, in the presence of 750 nM NBQX. C, Spike-counts during preferred (filled circles) and null (open squares) direction stimuli plotted against time. Gray box indicates duration of NBQX application. D, Relative reduction of preferred direction spiking. Bars are normalized to maximal control response for ON and OFF responses. Gray bars indicate the suppression of the preferred direction spiking responses. Error bars equal standard deviation.

In an effort to maximize the specificity of the NBQX effects, we first tested a range of concentrations (250 nM, 500 nM, 750 nM, 1 µM, 2 µM) to determine the lowest effective dose that disrupted directional responses in the DSGC. We found that the directionality of spiking responses in DSGCs could be reliably eliminated (19 of 19 cells) by 750 nM NBQX. To quantify the strength of the directional tuning in the DSGC we calculated the directional selectivity index (DSI) in the presence and absence of 750 nM NBQX (see Methods). In close agreement with previous estimates in rabbit [6], [20], DSI measured from spike responses averaged 0.52±0.12 for the ON response and 0.55±0.15 for the OFF response in control conditions (Figure 4A, N = 6). Application of 750 nM NBQX increased the number of null direction spikes and decreased the number of preferred direction spikes, so that the number of spikes elicited was independent of stimulus direction (Figure 4C). The average DSI measured in the presence of 750 nM NBQX was reduced to 0.07±0.02 for the ON response and 0.06±0.28 for the OFF response (Figure 4B, n = 6). The effect of NBQX was reversible and repeatable (Figure 4C). Higher concentrations of NBQX also blocked DS signals in the DSGCs, but produced progressively more suppression of the spiking responses (data not shown).

**Figure 5:**
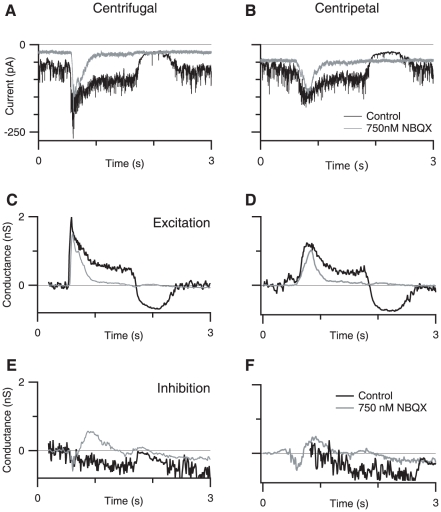
NBQX does not alter directionality of SBAC currents. A,B, Control currents (black traces) and currents recorded in the presence of 750 nM NBQX (black traces), in response to centrifugal (A) and centripetal stimuli (B). C,D, Excitatory conductance for centrifugal (C) and centripetal (D) stimulation in control (black traces) and 750 nM NBQX (gray traces).E,F, Inhibitory conductance for centrifugal (E) and centripetal (F) stimulation in control (black traces) and 750 nM NBQX (gray traces).

Is the loss of directional signals due to a reduction in the excitatory drive to SBACs as Cohen & Miller [21] suggested or due to a block of the directional mechanism in the SBAC as Hausselt *et al.* hypothesize? To test this idea, we measured light-evoked synaptic conductances in SBACs in the presence of 750 nM NBQX (Figure 5). We found that 750 nM NBQX blocked a net inward current of 13.9±8.9 pA and decreased the root mean square membrane current from 10.7±7.9 pA to 2.5±2.7 pA, consistent with the blockade of a sustained AMPA mediated input to the SBAC (Figure 5 A & B; p<0.005, n = 8). By examining the excitatory (Figure 5C) and inhibitory conductance (Fig 5D) we observed that NBQX completely blocked the sustained component of the light response for both centrifugal and centripetal motion. The transient initial component of the light response was slightly reduced; however, for centrifugal stimulation this reduction was variable and failed to reach significance (19.1±23% reduction, p = 0.058, n = 8). For centripetal motion the percent reduction was greater and reached significance (34.4±21.6% reduction, p = 0.012, n = 8). Because the reduction was larger for centripetal stimulation, this increased the directional difference of the SBAC response in the presence of NBQX.

**Figure 6:**
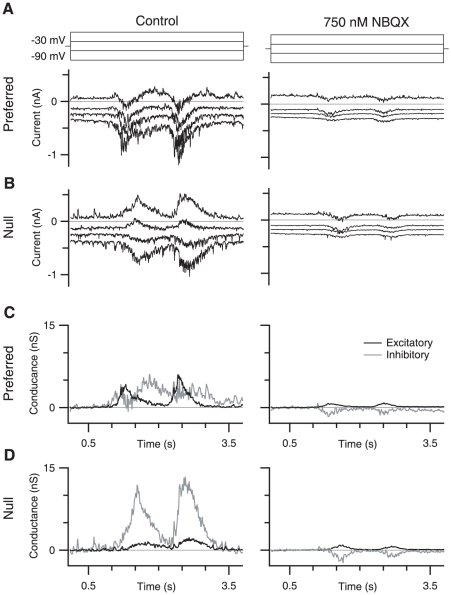
NBQX blocks inhibitory and most excitatory input to the DSGC. A, Current recordings during preferred direction stimulation at a series of command potentials between −90 and −30 mVs, in control (left traces) and in the presence of 750 nM NBQX (right traces). B, Current recordings for null direction stimulation. C, Excitatory (black trace) and inhibitory (gray trace) conductance for preferred direction stimulation in control (left traces) and in the presence of 750 nM NBQX (right traces). D, Same as C, but for null direction stimulation evoked conductance.

Thus, NBQX does not eliminate directional signals in the SBACs, nor does it completely suppress excitatory inputs, but are these residual inputs large enough to maintain the inhibitory drive to the DSGCs? To answer this question, we measured excitatory and inhibitory synaptic conductance in the DSGC in the presence of 750 nM NBQX (Figure 6). During null-direction stimulation, the integral of the inhibitory conductance was reduced by 96±8% & 97±5% for ON and OFF responses respectively (Figure 6 D, n = 9). The corresponding reductions for preferred direction stimulation were 96±13% & 95±14 for ON and OFF responses respectively (Figure 6 C). The larger error for preferred direction measurements was due to the smaller absolute magnitude of the preferred direction inhibition in control conditions. Thus while NBQX blocks the transmission of the directional signal from the SBAC to the DSGC it does not block directional responses in the SBAC.

Our results indicate that, contrary to recent reports, neither GABAergic inhibition nor tonic excitatory inputs are required to generate directional excitatory currents in the SBAC [12], [13]. Other explanations for the generation of directional signals in the SBAC that are independent of inhibition, have suggested that the small diameter of the SBAC dendrites results in electrical isolation of the soma from the dendritic terminals [22], [23], [24], [25], [26]. This notion raises the possibility that currents might be asymmetrical due to differences in the activation of voltage-gated channels in the proximal versus distal dendrites [27]. We wanted to examine this possibility by blocking voltage-gated currents that could be shaping directional responses.

### TTX-resistant sodium channels boost directional synaptic input to the SBAC

Previous reports have suggested that directional responses in SBACs could be enhanced by voltage-gated ion channels in the SBAC dendrites [13], [25]. There are reports of both voltage-gated calcium and sodium currents in SBACs; however, the existence of TTX-sensitive channels in SBACs has been disputed [13], [17], [18], [28], [29], [30], [31]. More recently, a systematic study of the voltage-gated currents in mouse SBACs found evidence for N, P and Q type calcium currents, but did not find evidence for TTX sensitive sodium currents [32]; however, they did not rule out the possibility of tetrodotoxin-resistant voltage-gated sodium channels (TTX-R VGSC), and a subsequent immunohistochemical study suggests that TTX-R VGSC (Na_V_ 1.8) are expressed in SBACs [33]. In light of these previous studies, we examined the effect of ambroxol hydrochloride, a selective TTX-R VGSC blocker [34], on SBAC light responses to determine whether TTX-R sodium currents contribute to directional signals.

**Figure 7:**
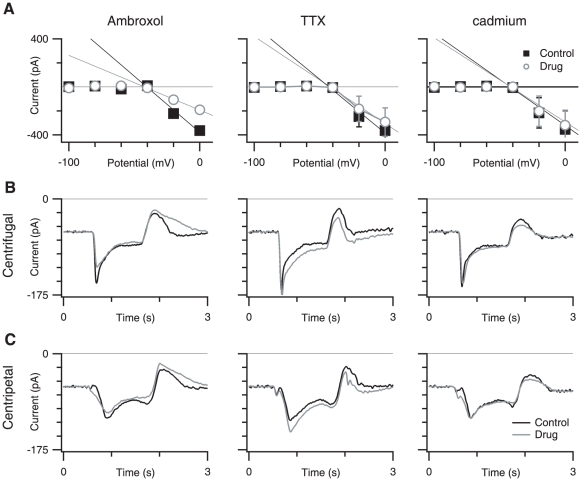
Ambroxol reduces SBAC AI, but TTX or cadmium does not. A, (top row) shows the average IV relation measured 1.2 ms after the command step to voltages between −100 and 0 mV, under control conditions (closed squares) and drug (open circles). Error bars show standard error. The linear portion of the IV has been subtracted leaving the negative slope portion of the IV (see Methods). The solid lines indicate the best-fit line between −40 and 0 mV. A shows 300 µM ambroxol hydrochloride (n = 3), B shows 500 nM TTX (n = 6), and C shows 25 µM cadmium (n = 5). B and C, (second and third rows) show the average filtered (25 Hz cutoff Gaussian) current traces in response to centrifugal and centripetal stimulation, respectively in control conditions (black trace), and drug treatment (gray trace). Left column shows 300 µM ambroxol hydrochloride (n = 3). Middle column shows. 500 nM TTX (n = 5). Right column shows 25 µM cadmium (n = 6).

We applied a series of depolarizing voltage steps and measured the IV relation between −100 and 0 mV. When the linear component of the membrane current between −100 and −40 mV was subtracted (see Methods), the IVs displayed a negative slope between −40 and 0 mV, consistent with the activation of an inward voltage-gated current (Figure 7A). This negative conductance appeared to be mediated in part by TTX-R VGSC because application of 200–500 µM ambroxol reduced the negative conductance between −40 and 0 mV from −3.51±0.70 to −1.74±0.37 nS (P = 0.015, n = 3).

To assess the role of TTX-R channels in the SBAC response to light stimuli, we applied ambroxol during centrifugal and centripetal stimulation. Ambroxol decreased the peak amplitude of currents elicited by centrifugal stimulation by 48±13% (p = 0.002, n = 6), but had no effect on the sustained component of the light response. The peak amplitude of the centripetal response was reduced by 37±20% (p = 0.008, n = 6). This larger reduction in centrifugal peak amplitude meant that ambroxol significantly reduced the directional difference between centrifugal and centripetal peak amplitudes by 64±10% (p = 0.002, n = 6), with the result that the AI for amplitude was significantly decreased from 0.26±0.07 to 0.19±0.08 (p = 0.008, n = 6, Figure 7A). Despite the significant reduction in the asymmetry, the centrifugal and centripetal amplitudes were still significantly different from each other (p = 0.001). These results indicate that TTX-R sodium channels contribute to generating the directional response in SBACs; however, they cannot completely account for directional selectivity.

In light of past differences regarding the presence of TTX-sensitive channels in SBACs, and to control for possible non-specific block of TTX-sensitive VGSCs by ambroxol, we tested whether blocking the TTX-sensitive VGSCs directly would affect the directional asymmetry in SBACs. To this end, we measured SBAC responses in the presence of 500 nM TTX (Figure 7B). TTX had no significant effect on voltage-activated currents in the SBAC. In contrast to ambroxol, TTX increased the peak amplitude of the SBAC responses by 15±11% for centrifugal and 29±20% for centripetal stimuli (p = 0.027 and 0.023, respectively, n = 5). Because both centrifugal and centripetal response amplitudes were changed in the same direction, TTX did not have a significant effect on the directional differences or the AI. These data show that block of TTX-sensitive VGSCs cannot account for the effects seen with ambroxol. The most parsimonious explanation for the increase of the SBAC response amplitude by TTX is that TTX suppresses the activity of spiking amacrine cells which make inhibitory contacts with bipolar cell terminals [35], [36]. (Figure 7B).

Previous work has shown that voltage-gated calcium channels (VGCCs) are present in SBACs [32], [37] and it is possible that these may contribute to the directional asymmetry in the SBACs [13], [25]. Because high concentrations of cadmium that completely block VGCCs also abolish synaptic transmission and light responses in the retina, we needed to test the effects of low concentrations of cadmium on the SBAC responses. Previous work has shown that low concentrations of cadmium selectively block direction selectivity without abolishing light responses [13], [30]. When we measured SBAC responses in the presence of 25–30 µM cadmium we did not see any change in voltage-activated currents in the presence of this concentration of cadmium, nor could we resolve any differences in response amplitudes, rise times, or the AI (Figure 7C) of the light evoked SBAC response. We did not examine the effects of higher concentrations of cadmium, because non-specific blockade of synaptic transmission confound the interpretation of these experiments (data not shown).

## Discussion

In agreement with our current measurements, previous work has established that both voltage and current responses in SBAC soma are direction-selective [11], [12], [13]; however, our data cannot support either of the current models for SBAC direction selectivity [11], [12], [13]. In addition, we show that TTX-sensitive VGSCs do not contribute to generating SBAC directional signals; however, the data suggests that TTX-resistant channels may play a modest role in enhancing these directional signals.

### Models of SBAC direction selectivity

One hypothesis with recent experimental support is that direct inhibitory input from reciprocal interactions between neighboring SBACs is responsible for generating directional asymmetries [12]. According to this hypothesis, surround inhibition mediated by adjacent SBACs will produce directional asymmetries in the magnitude of inhibitory inputs during centrifugal and centripetal stimulation. However, our results show that directional responses in SBACs are dominated by directional excitation, with little detectable inhibitory input. This difference cannot be ascribed simply to a difference in technique because, apart from a difference in the strain of rabbits (the previous study used NZ white rabbits while we have used Dutch-belted), the methods are essentially the same. We cannot discount the possibility that a difference in light adaptation level might cause some difference in the amount of inhibition seen in the SBACs, although this will need further systematic analysis to test.

The lack of inhibition in the SBACs is unexpected given the reciprocal inhibition results cited above. The SBAC dendrites are thin, and the distal dendrites are likely to be electrotonically distant from the recording electrode. Therefore, the inhibitory input seen at the soma will under-estimate the actual synaptic conductance, however, such errors will be similar in both studies and are unlikely to account for the difference. Moreover, we occasionally observed a light-evoked inhibitory conductance in the SBACs when we applied the AMPA/kainate antagonist NBQX (Figure 5D), indicating that inhibition can be resolved in the SBACs under appropriate conditions. It is also worth noting that under identical adaptation conditions we record robust directional responses in DSGCs (Figure 4A), indicating that the lack of large reciprocal GABA currents in SBACs does not preclude directional responses in the DSGCs. Furthermore, directional calcium responses observed in SBACs, are independent of GABA_A_ receptor activity Hausselt *et al.* [13], and consistent with this result, we observed directional excitatory inputs when GABA_A_ receptors were blocked. Thus, results presented here and previously, suggest that reciprocal inhibition is not necessary to generate directional responses in SBACs or DSGCs.

After blocking inhibition, Hausselt *et al.* [13] found that directional signals in SBACs were modulated by SBAC membrane potential, and suggested a role for a voltage-dependant non-linearity. Based on measurements, and compartmental modeling, they proposed that directional discrimination relies on an interaction between voltage-gated calcium channels in the distal dendrites and a dendrite-to-soma voltage gradient created by a tonic excitatory glutamatergic input [17], [18]. We tested the hypothesis that the tonic excitatory input is necessary for directional responses in SBACs by blocking the tonic glutamatergic input, which should dissipate the dendritic voltage gradient proposed by Hausselt *et al.* [13]. Although NBQX suppressed a tonic inward current, and blocked the sustained component of the light-response, the directional asymmetry in excitatory responses of SBACs remained robust. Paradoxically, NBQX did abolish directional responses in the DSGCs, as was observed previously [21], even though the SBACs remained directional in the presence of NBQX. One explanation for this apparent contradiction comes from the observation that NBQX blocks a tonic depolarizing current in the SBACs. Perhaps blocking this tonic excitation hyperpolarizes the SBACs so that that the light-evoked synaptic potentials fail to reach threshold for synaptic release, thereby blocking forward transmission of the directional signal from the SBAC to the DSGC. In summary, our data is in agreement with previous studies in showing marked directional asymmetries in the responses of SBACs, however, the results also raise some interesting discrepancies in the current models explaining the mechanism generating the directional signals that will require further study to fully resolve.

### A possible role of TTX-Resistant sodium channels in SBAC direction selectivity

Other models of SBAC direction selectivity have focused on the observation that SBAC dendrites are thin and electrotonically distant [23], [24], [25], [38], which could allow for an asymmetrical activation of voltage-gated conductances. Experimental [13], [30] and theoretical [25] studies, have proposed a role for voltage-gated calcium channels in generating directional responses in the SBACs. Based on extracellular recordings from ganglion cells in the presence of Cd^2+^ (70–100 µM) and selective conotoxins, Jensen [30], [39] suggested that P or Q type calcium channels were necessary for generating directional responses. Subsequently, Hausselt *et al.* [13] found that low concentrations of cadmium (10 µM) reduced SBAC DS without blocking synaptic transmission; yet they found that conotoxins did not have any significant effect on the SBAC directional asymmetry, short of blocking all light-evoked synaptic input to the cell. However, cadmium can also block TTX-resistant voltage-gated sodium channels [40], [41], [42] raising the possibility that previous results with low concentrations of cadmium may have implicated TTX-R VGSCs.

Our pharmacological results along with recent anatomical evidence suggesting the presence of Na_V_ 1.8 in SBACs [33], indicate that the tetrodotoxin-resistant sodium channel type Na_V_ 1.8 could contribute to generating directional signals in SBACs. Na_V_ 1.8 is an unusual sodium channel both in its pharmacological profile as well as its activation and inactivation kinetics. While it is largely insensitive to the classic sodium channel blockers TTX and QX-314, it is blocked by ambroxol hydrochloride with sensitivity three times greater than TTX-sensitive channels [34]. To control for effects of ambroxol on TTX-sensitive channels we also examined the effect of TTX on directional signals in the SBAC. TTX did alter the shape of the SBAC light responses presumably due to effects on voltage-gated sodium channels located presynaptically to the SBAC, possibly at bipolar cell terminals [43], [44], or by its effects on spiking interneurons that synapse onto bipolar cell terminals [45]. Regardless, TTX did not have any effect on SBAC direction selectivity, indicating that the effect of ambroxol was specific to TTX-resistant sodium channels. Because there is no evidence for TTX-resistant sodium channels on bipolar cell terminals, or other interneurons [33] we conclude that the effects of ambroxol on SBAC directional signaling were due to blockade of TTX-resistant sodium channels on the SBAC.

Na_V_ 1.8 channels are also kinetically distinct from TTX-sensitive sodium channels as they have slower activation and inactivation kinetics, a more depolarized activation threshold, and more depolarized steady-state activation and inactivation curves. The inactivation time constant at 0 mV is approximately 5 ms about ten fold slower than for typical TTX sensitive currents. Na_V_ 1.8 also has a more depolarized half maximal activation value of approximately −15 mV compared to −27.6 to −40 mV for TTX-sensitive currents [46], [47], [48]. While the activation range of these channels is more depolarized than the voltages we recorded from the soma of the SBAC, many studies suggest that the dendrites of the SBAC are likely more depolarized, which may allow for activation of Na_V_ 1.8 channels [13] [49], [50]. The relatively slow kinetics of the TTX-R sodium channels seems well suited to a role in the visual system, where the kinetics of the synaptic inputs are also slow, relative to other brain regions. In addition, the comparatively positive activation potential of Na_V_ 1.8 gains intriguing significance because outwardly-rectifying potassium channels are expected to limit depolarization of the SBACs to sub-threshold voltages [26]. Moreover, Ozaita *et al.* [26] found evidence for a gradient of these potassium channels in the SBAC with the highest levels found in the soma and progressively lower levels in the dendrites. Perhaps the lower potassium channel density and higher local input resistance in the distal dendrites allows the excitatory inputs to reach threshold for the TTX-R sodium channels.
